# Supplementary material for: Enhanced IGFL1 translation in response to IL-1β is controlled by distinct 3’UTR elements
Source: PLoS One. 2026 Jun 25;21(6):e0342288. doi: 10.1371/journal.pone.0342288 (PMC13298950; doi:10.1371/journal.pone.0342288)
Supplement: S1 File — S1 Table. Primers used in this study; S1 Fig. IGFL1 mRNA expression upon TNF-α, IL-6, IL-10, and IL-1β stimulation; S2 Fig. Effect of IL-1β on CXCL8 mRNA expression, mRNA stability, and translation; S3 Fig. Predicted structures of the deletion constructs; S4 Fig. Translation-regulatory activity of the isolated G-rich region of the IGFL1 3’UTR; S5 Fig. RBP prediction using ATtRACT. (PDF) [file pone.0342288.s001.pdf]

Supporting information

S1 Table. Primers used in this study.

primer	forward	reverse
GAPDH (qPCR)	TGCACCACCAACTGCTTAGC	GGCATGGACTGTGGTCATGAG
IGFL1 (qPCR)	CACAAGAGATGTGGGGACAAG	CACTGCGACAAAGCCTGTCA
CXCL8 (qPCR)	TCCTGATTTCTGCAGCTCTGT	AATTTCTGTGTTGGCGCAGT
IGFL1 (5'UTR cloning)	CTCACTATAGGCTAGCCCCTCCTCACTCCA	AGCCATGGTGGCTAGGGCTCTGGGTGGTTG
IGFL1 (full length 3'UTR cloning)	<u>CAGTAATTCTAGGCGTGGAACATCAGGGGAACGATGA</u>	TTGCGGCCAGCGGCCTTCATTTATAAAGTACATAAATTT
1-309 construct	<u>CAGTAATTCTAGGCGTGGAACATCAGGGGAACGATGA</u>	TTGCGGCCAGCGGCCAGCCAGCCTTGGGGTGGTG
1-279 construct	<u>CAGTAATTCTAGGCGTGGAACATCAGGGGAACGATGA</u>	TTGCGGCCAGCGGCCTTGGCCATAGGGGTCATCA
1-247 construct	<u>CAGTAATTCTAGGCGTGGAACATCAGGGGAACGATGA</u>	TTGCGGCCAGCGGCCTCCCTGGGCCCCCATCAG
1-206 construct	<u>CAGTAATTCTAGGCGTGGAACATCAGGGGAACGATGA</u>	TTGCGGCCAGCGGCCAATTCTAGAAGGGGACCTGTGGG
1-103 construct	<u>CAGTAATTCTAGGCGTGGAACATCAGGGGAACGATGA</u>	TTGCGGCCAGCGGCCCCAAACAGCCACTCAGCATCC
Δ207-247 construct insert 1	ACGAGCAGTAATTCTAGGCGTGGAACATCAGGGGAACGATGAC	GGGTTCAGAGAATTCTAGAAGGGGACCTGTGGG
Δ207-247 construct insert 2	TTCTAGAATTCTCTGAACCCTCCTGATGA	TTTTATTGCGGCCAGCGGCCTTCATTTATAAAGTACATAAATTT
G238C construct	GCGTGTGCTGATGGGCGCCCAGGGACTCTGA	TCAGAGTCCCTGGGCGCCCATCAGCACACGC
G245T construct	CTGATGGGGGCCCAGTGACTCTGAACCCTCC	GGAGGGTTCAGAGTCACTGGGCCCCCATCAG
G238C_ C277G construct	GATGACCCCTATGGCGAACATCAACCCGGCA	TGCCGGGTTGATGTTCGCCATAGGGGTCATC
G245T_ C269A construct	ACCCTCCTGATGACCACTATGGCCAACATCA	TGATGTTGGCCATAGTGGTCATCAGGAGGGT
Δ235-239 construct	GACAGCATGAGATGCGTGTGCTGATCCCAGGGACTCTGAACCCTCCTGAT	ATCAGGAGGGTTCAGAGTCCCTGGGATCAGCACACGCATCTCATGCTGTC
Δ243-247 construct	GAGATGCGTGTGCTGATGGGGGCCCCTCTGAACCCTCCTGATGACCCCTA	TAGGGGTCATCAGGAGGGTTCAGAGGGGCCCCCATCAGCACACGCATCTC
Δ235-247 construct	GACAGCATGAGATGCGTGTGCTGATCTCTGAACCCTCCTGATGACCCCTA	TAGGGGTCATCAGGAGGGTTCAGAGATCAGCACACGCATCTCATGCTGTC

Underlined primers are used in multiple assays.

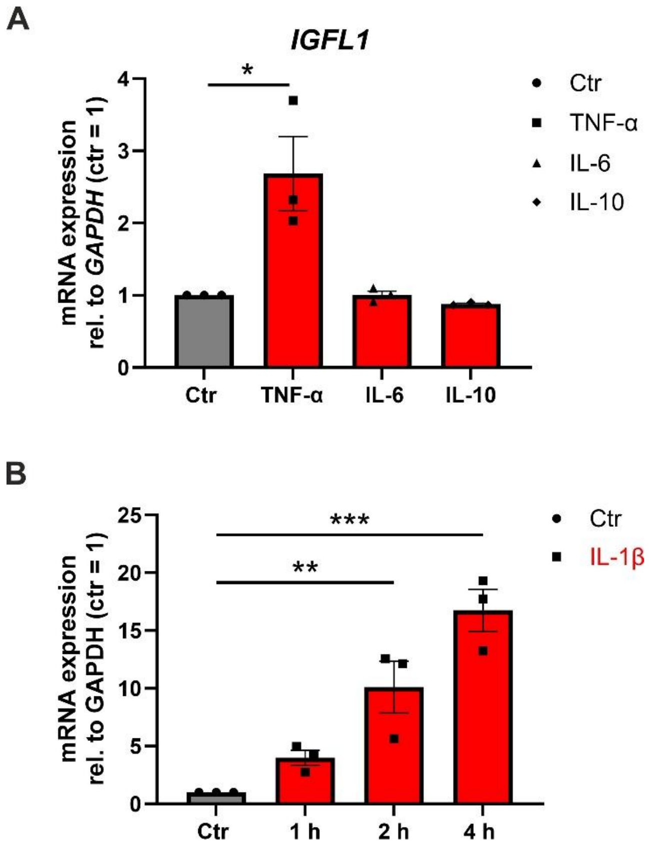

S1 Fig. *IGFL1* mRNA expression upon TNF-α, IL-6, IL-10, and IL-1β stimulation. (**A**) MCF7 cells were treated with TNF-α, IL-6 and IL-10 (50 ng/mL) for 4 h. (**B**) MCF7 cells were treated with IL-1β (50 ng/mL) for 1, 2, 4 h. *IGFL1* mRNA expression was measured by RT-qPCR and normalized to *GAPDH* expression (n = 3). Data were statistically analyzed using two-way ANOVA with Dunnett's multiple comparisons test; * $p < 0.05$, ** $p < 0.01$, *** $p < 0.001$ compared to respective untreated controls.

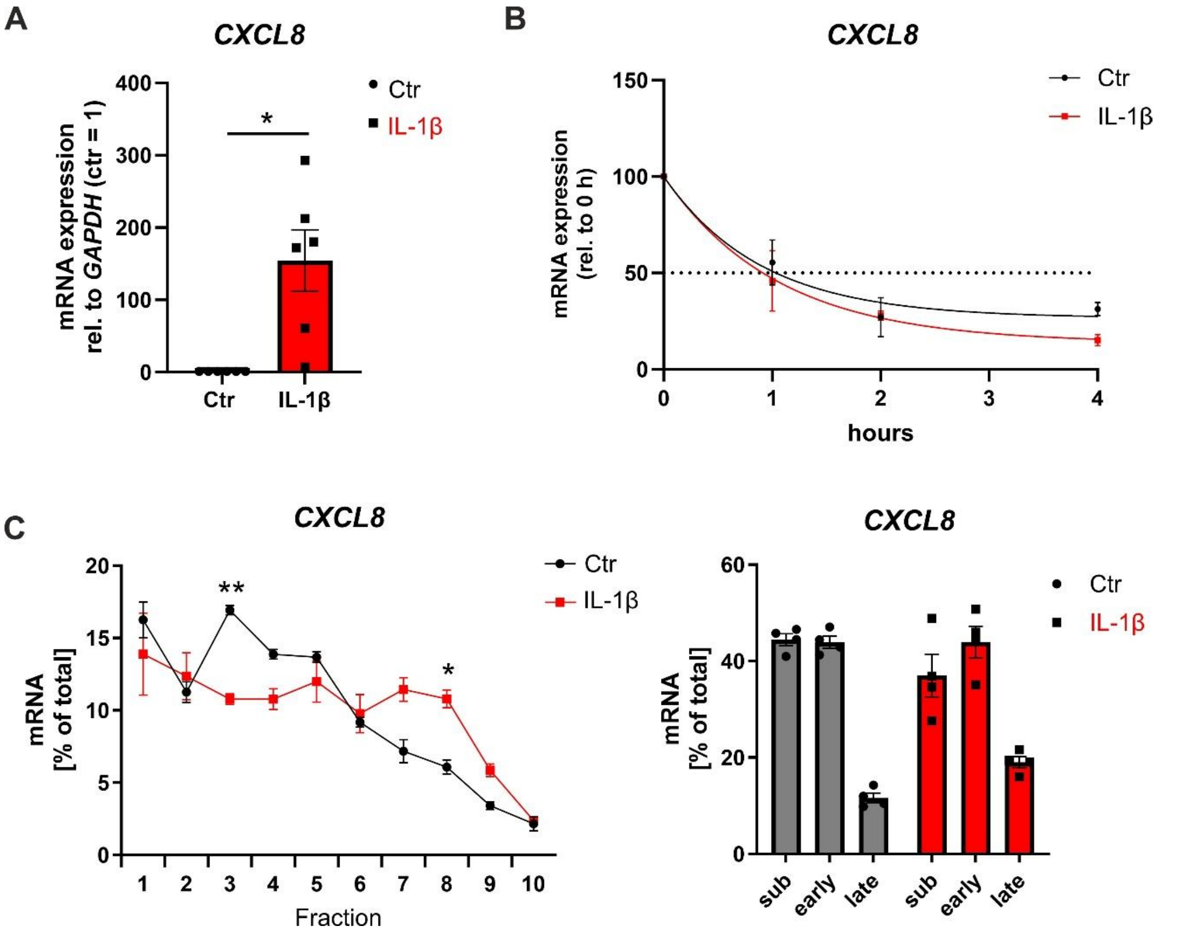

S2 Fig. Effect of IL-1β on *CXCL8* mRNA expression, mRNA stability, and translation. MCF7 cells were treated with IL-1β (50 ng/mL) for 4 h. (**A**) *CXCL8* mRNA expression was measured by RT-qPCR and normalized to *GAPDH* expression (n = 6). (**B**). Transcription was blocked by the addition of actinomycin D (10 µg/mL) at the end of the IL-1β treatment and mRNA expression was followed for up to 4 h by RT-qPCR analyses (n = 3). (**C**) Translational status of *CXCL8* was assessed by polysomal fractionation analysis. *CXCL8* mRNA (distribution across the gradients was analyzed by RT-qPCR (n = 4). The distribution across all the fractions and for sub, early and late polysomal fractions are showed in the left and right panel, respectively. All data were presented as means ± SEM and statistically analyzed using paired t-test (**A**) or two-way ANOVA with Šídák's multiple comparisons test (**B-C**); * $p < 0.05$, ** $p < 0.01$, compared to respective untreated controls.

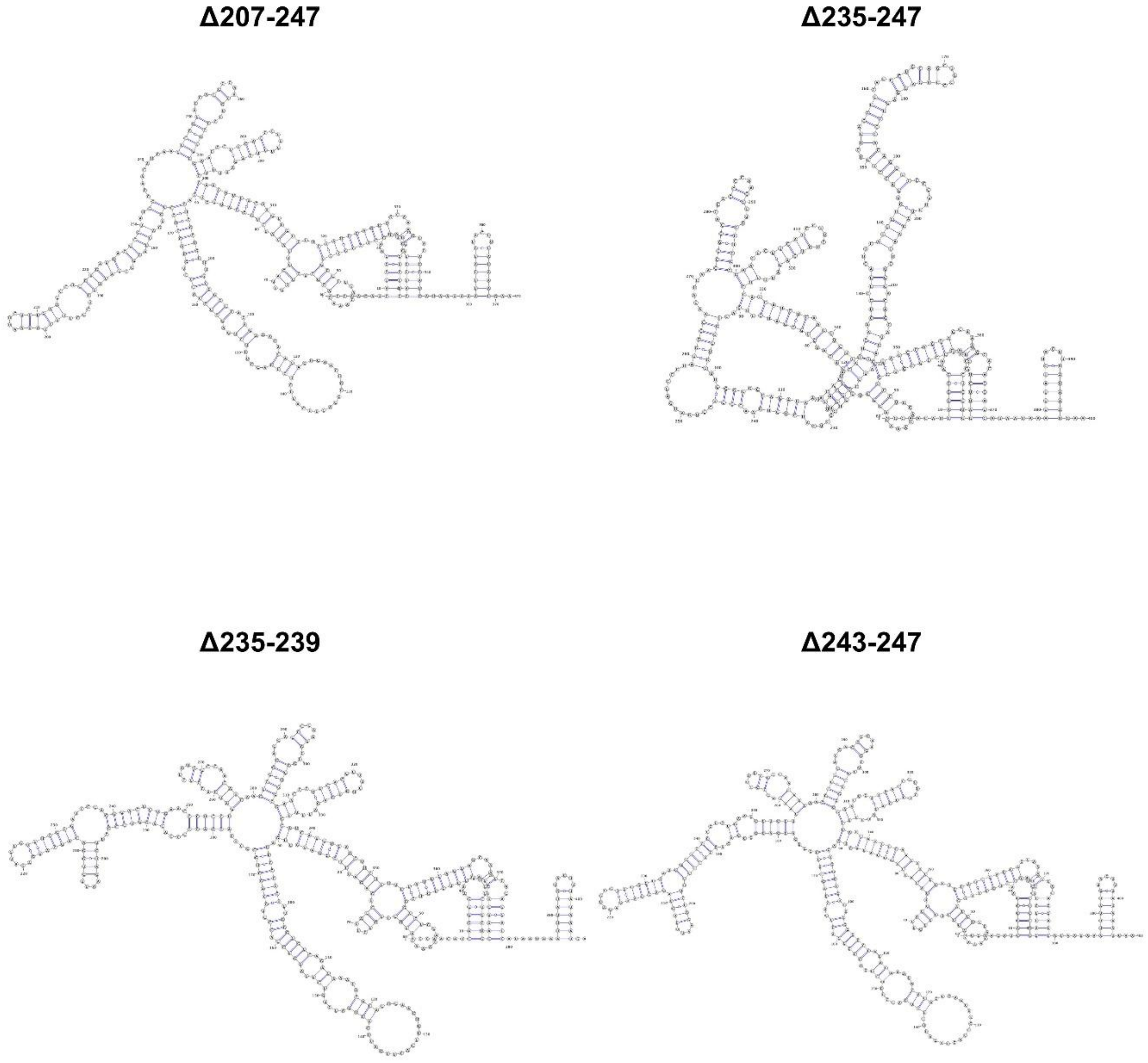

S3 Fig. Predicted structures of the deletion constructs. The depicted structure were generated by VARNA [1].

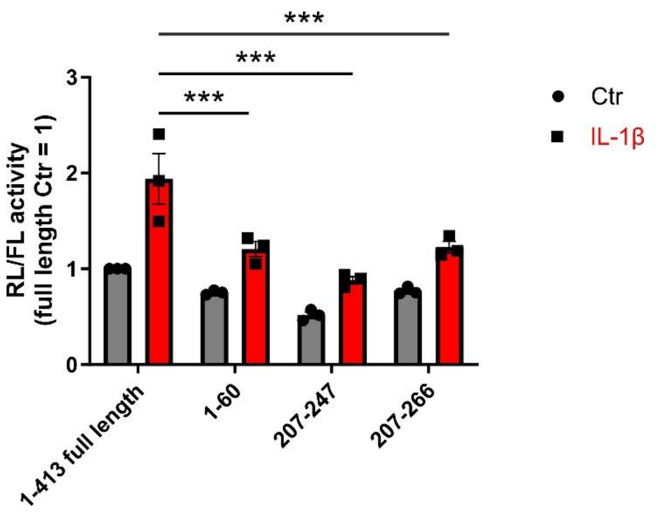

S4 Fig. Translation-regulatory activity of the isolated G-rich region of the *IGFL1* 3'UTR. MCF7 cells were transfected with the vector containing either the full length or indicated regions of the *IGFL1* 3'UTR. *Renilla* (RL) and *firefly* luciferase (FL) activities were determined 48 h after the transfection with or without treatment with IL-1β (50 ng/mL) during the last 4 h. Data were normalized to the full length 3'UTR control vector, presented as means ± SEM (n = 3), and statistically analyzed using two-way ANOVA with Dunnett's multiple comparisons test; *** $p < 0.001$ compared to the IL-1β-treated full length 3'UTR vector.

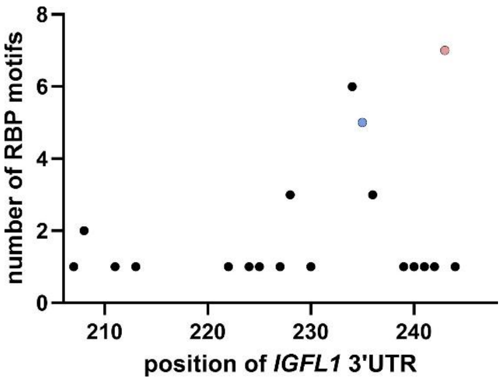

S5 Fig. RBP prediction using ATtRACT. Graphical representation of the RBP motif results using ATtRACT [2]. The number of RBP binding motifs for each position of the region 207-247 of *IGFL1* is depicted. Blue and red dots represent the GGGGG motif at position 235-239 and the AGGGA motif at position 243-247, respectively.

References

1. Darty K, Denise A, Ponty Y. VARNA: Interactive drawing and editing of the RNA secondary structure. Bioinformatics. 2009 Aug 1;25(15):1974–5.
2. Giudice G, Sánchez-Cabo F, Torroja C, Lara-Pezzi E. ATtRACT—a database of RNA-binding proteins and associated motifs. Database (Oxford). 2016 Apr 7;2016:baw035.
